# DNA-PKcs inhibition sensitizes glioblastoma to radiotherapy through reprogramming of tumor cell states and immune microenvironment cell types

**DOI:** 10.21203/rs.3.rs-9657955/v1

**Published:** 2026-06-05

**Authors:** David Raleigh, S. John Liu, Nazanin Majd, Christopher Zou, Harrsha Congivaram, Hinda Najem, Mark Youngblood, I-Chang Wang, Sasha Sengelmann, Krish Raval, Jialong Jiang, Kyla Foster, Tim Casey-Clyde, Joanna Pak, Sixuan Pan, Emily Payne, Katie Lin, Harish Vasudevan, Matt Thompson, Tomoko Ozawa, Kanish Mirchia, Mitchel Berger, William Weiss, Amy Heimberger, Johh de Groot, Luke Gilbert

**Affiliations:** University of California San Francisco; University of California San Francisco; University of Texas MD Anderson Cancer Center; University of California San Francisco; Northwestern University; Northwestern University; Northwestern University; University of California San Francisco; University of California San Francisco; University of California San Francisco; Cal Tech; University of California San Francisco; University of California San Francisco; UCSF; University of California San Francisco; University of California San Francisco; University of California San Francisco; University of California San Francisco; Cal Tech; University of California San Francisco; Univeristy of California San Francisco; University of California San Francisco (UCSF); UCSF; Northwestern University; University of California San Francisco; Arc Institute/UCSF

## Abstract

Glioblastoma is the most common malignant brain tumor in adults, and radiotherapy (RT) is the most effective postoperative treatment for patients with glioblastoma. Nevertheless, glioblastoma recurrence after treatment is nearly universal, and cell state plasticity and intratumor heterogeneity underlie glioblastoma evolution and resistance to treatment. Here we integrate in vitro genome-wide CRISPR interference screens and in vivo perturb-seq in preclinical models with single-nucleus and spatial transcriptomic sequencing of human tumors to identify therapeutic vulnerabilities that overcome glioblastoma resistance to RT. Gene regulatory network modeling identifies DNA-PKcs as a RT-sensitizing target in glioblastoma cells in vitro and in vivo. Small molecule inhibition of DNA-PKcs plus RT improves survival and reprograms tumor cell states and immune microenvironment composition compared to DNA-PKcs inhibition or RT monotherapy. Bioinformatic and imaging analyses of patient-matched glioblastoma samples before and after DNA-PKcs inhibition and RT show that combination therapy drives inflammatory gene expression programs in tumor cells that recruits pro-inflammatory myeloid cells to the tumor microenvironment. Using this framework to inform rational sequential therapy in preclinical models, we show that immunomodulation in response to genomic stress after DNA-PKcs inhibition and RT primes glioblastoma for response to cGAS/STING activation. These data show that DNA-PKcs modulates tumor cell states and immune microenvironment cell types to drive resistance to RT in glioblastoma, and that targeting DNA-PKcs sensitizes glioblastomas to RT and cGAS/STING activation.

Glioblastoma is the most common primary malignant brain tumor in adults^1^, and patients have a median overall survival of 14–18 months despite treatment with surgery, chemotherapy, and radiotherapy (RT)^2–5^. Glioblastoma exhibits remarkable cellular, genetic, and epigenetic heterogeneity^6–13^, and resistance mechanisms in malignant and tumor microenvironment (TME) cells represent significant barriers to improving survival for patients^3,6,7,14–16^. Clinical data suggest that RT is the most effective postoperative therapy for glioblastoma^2^, but current approaches to increase the efficacy of RT are limited to alkylating chemotherapies^3,17,18^. Thus, there are unmet needs for new techniques that elucidate therapeutic vulnerabilities to overcome glioblastoma resistance to treatment, and for new combination or sequential therapies to improve outcomes for patients.

CRISPR/Cas9 functional genomic approaches have transformed understanding of how genetic perturbations impact myriad cell types and cell states^19–25^, and high throughput functional genomic screens have identified promising therapeutic targets in preclinical models of glioma^26–31^. However, prior functional genomic studies in glioblastoma have relied on *in vitro* cell culture models that do not recapitulate the *in vivo* heterogeneity, physiology, or TME of glioblastomas in patients. To address this, we recently developed a multiplex *in vivo* CRISPR interference (CRISPRi) platform with single-cell RNA sequencing readout (perturb-seq) to perform genetic screens in tumor and TME cells in glioblastoma *in vivo* preclinical models^32^. By leveraging intracranial convection enhanced delivery (CED) of sgRNA libraries into patient-derived xenograft (PDX) or allograft models of glioblastoma in mice, this functional genomic approach for *in vivo* perturb-seq can be performed in tumors with a TME to identify therapeutic vulnerabilities that can be exploited to reprogram treatment resistance pathways^32^. More broadly, our approach for *in vivo* perturb-seq enables the identification and definition of molecular pathways that originate in perturbed cells that can modulate the surrounding TME.

Here we integrate genome-wide CRISPRi screens and *in vivo* perturb-seq in glioblastoma preclinical models to define clinically actionable targets that increase the efficacy of RT, thereby nominating new therapeutic strategies for this fatal disease. Gene regulatory network (GRN) analyses using dimension-scalable single-cell perturbation integration network (D-SPIN) inference^33^ on *in vivo* perturb-seq data reveal that *PRKDC*, which encodes the catalytic subunit of DNA dependent protein kinase (DNA-PKcs)^34,35^, is a master regulator of multiple tumor cell intrinsic and immune microenvironment pathways that drive glioblastoma resistance to RT. We validate these findings using peposertib, a selective small molecule inhibitor of DNA-PKcs^36–39^, in glioblastoma preclinical models and in patient-matched newly diagnosed and recurrent glioblastoma samples from a phase 1 clinical trial of RT with concurrent peposertib (NCT04555577). Single-nucleus and spatial transcriptomic sequencing of patient samples reveal that DNA-PKcs inhibition and RT induce inflammatory signaling in tumor cells which drives immune priming and sensitizes glioblastoma preclinical models to cyclic guanosine monophosphate adenosine monophosphate synthase (cGAS) stimulator of interferon gene (STING) activation. These data establish a technical framework for elucidating response or resistance mechanisms to cancer treatment and shed light on new combination and sequential therapies to treat patients with glioblastoma.

## Functional genomic discovery and validation of radiotherapy response genes in glioblastoma preclinical models

**Figure 1:**
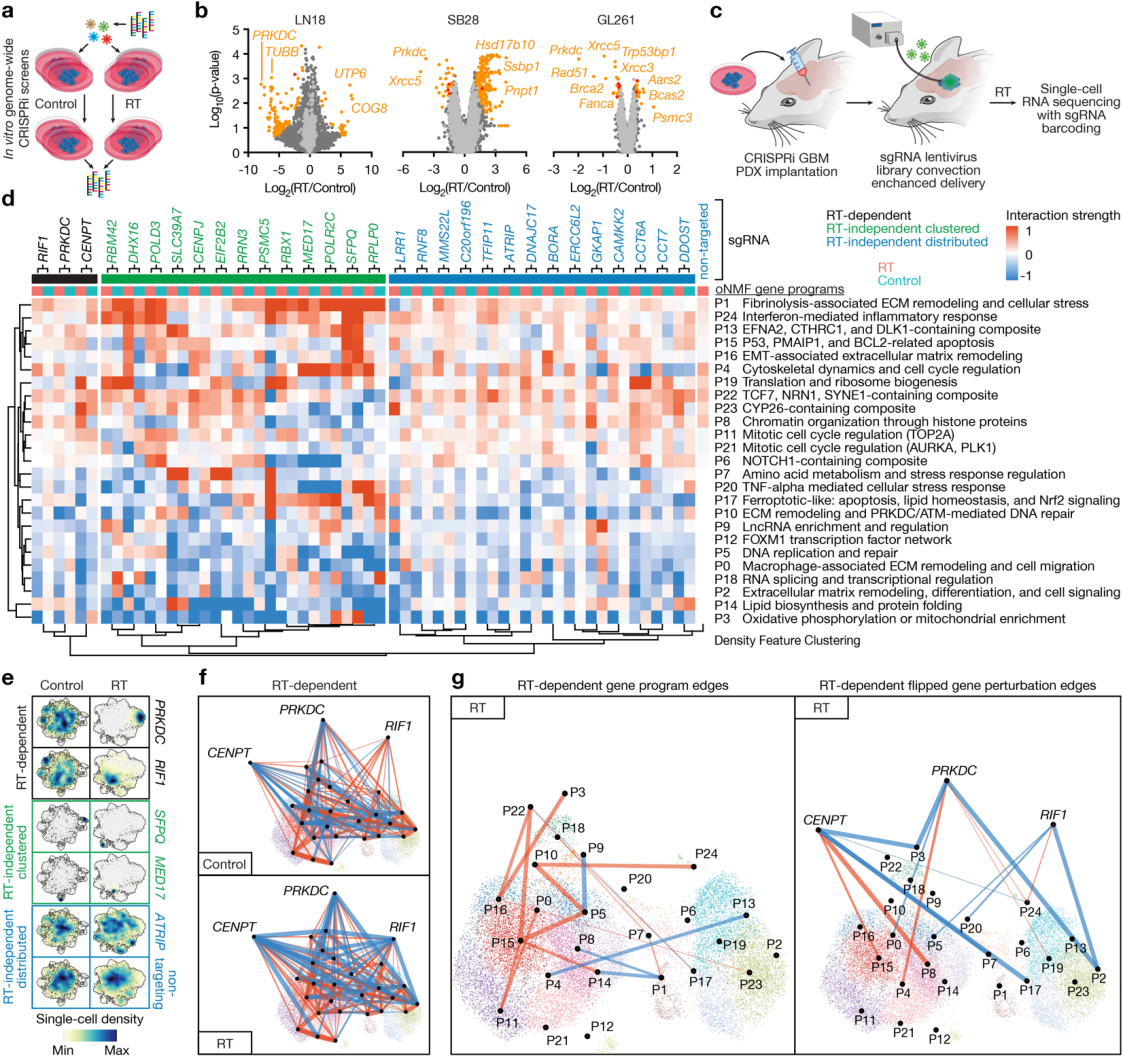
Functional genomic discovery and validation of radiotherapy response genes in glioblastoma preclinical models. **a**, Schematic for *in vitro* CRISPRi screens to identify RT response genes in glioblastoma cells using genome-wide (LN18, SB28) or druggable genome (GL261) sgRNA libraries. **b**, Volcano plots demonstrating CRISPRi screen enrichment phenotypes comparing RT at ~LD_50_ (2Gy × 2 daily fractions for LN18 and SB28, 2Gy × 5 daily fractions for GL261) to unirradiated control cultures. Genes driving RT sensitization upon suppression are on the left of each volcano plot, and genes driving RT resistance upon suppression are on the right of each volcano plot. Orange, targeted sgRNA hits meeting empirical false discovery rate < 2%. Red, non-targeted control sgRNA hits. Light gray, non-targeted control sgRNAs not scoring as a hit. Dark gray, targeted sgRNAs not scoring as a hit. **c**, Schematic for *in vivo* perturb-seq to define RT sensitizing genetic vulnerabilities in glioblastoma using convection enhanced delivery of sgRNA libraries and direct capture of sgRNAs in single-cells. **d**, Heatmap of interactions between single-cell sgRNA perturbations (columns) and gene expression programs (rows) from dimension-scalable single-cell perturbation integration network (D-SPIN) inference on *in vivo* perturb-seq data from GBM43 PDXs ± RT (2Gy × 2 daily fractions) across 16,114 single cells. 30 sgRNA perturbations are normalized to single-cells from unirradiated GBM43 PDXs expressing non-targeted control sgRNA *in vivo,* and paired control/RT treatments with each sgRNA perturbation are grouped in columns based on single-cell density feature patterns in LDA UMAP space. **e**, Representative LDA UMAPs of perturbation classes from **d** demonstrating distinct patterns of RT-dependent or RT-independent single-cell distributions. **f**, D-SPIN models of single-cell transcriptomes with RT-dependent perturbations in PDXs after control (top) or RT (bottom) treatments showing inferred activating (red) or inhibitory (blue) interaction vectors between sgRNA perturbations targeting *PRKDC, RIF1,* or *CENPT* and gene expression programs (P# nodes within each UMAP). Edge thickness is proportional to the strength of network interactions. **g**, D-SPIN models of inferred activating or inhibitory interactions between different gene expression programs that are significant with RT but not control treatments (left, P# defined in **d**) or between RT-dependent perturbations (*PRKDC, RIF1, CENPT*) and gene expression programs (right) that flip from inhibitory to activating (red) or from activating to inhibitory (blue) with RT compared to control treatment. Edge thickness is proportional to the strength of network interaction.

To identify genes that modify glioblastoma responses to RT, *in vitro* genome-wide CRISPR interference (CRISPRi) screens^40,41^ were performed using human (LN18) and mouse (SB28, GL261) glioblastoma cell lines in the presence or absence of RT at ~LD_50_ (Fig. 1a). Using an established screen threshold corresponding to an empiric false discovery rate of 1–2%^24,42^, 113 genes whose repression resulted in RT sensitization and 25 genes whose repression resulted in RT resistance were identified in the human glioblastoma cell line LN18 (Fig. 1b, Supplementary Table 1). In the murine SB28 glioblastoma cell line, 24 genes whose repression resulted in RT sensitization and 200 genes whose repression resulted in RT resistance were identified (Fig. 1b, Supplementary Table 1). Re-analysis of our CRISPRi screen data for RT response genes in GL261 mouse glioblastoma cells^32^ using the same empiric false discovery rate threshold^24,42^ identified 15 genes whose repression resulted in RT sensitization and 15 genes whose repression resulted in RT resistance (Fig. 1b, Supplementary Table 1). Ontology analyses demonstrated that RT sensitizing genes were enriched for DNA repair and DNA metabolic processes across all 3 glioblastoma cell lines (Extended Data Fig. 1a, b), and *PRKDC/Prkdc*, a regulator of non-homologous end joining (NHEJ)^34,35^, was one of the most RT sensitizing hits that was conserved across all 3 glioblastoma cell lines (Fig. 1b).

To validate these targets and define mechanisms underlying glioblastoma RT sensitization *in vivo*, perturb-seq was performed using CED of lentiviral sgRNA libraries into an intracranial glioblastoma PDX model, GBM43, which was engineered to express CRISPRi machinery^32^ (Fig. 1c). An mCherry-tagged dual sgRNA lentivirus library targeting 35 genes whose knockdown sensitized glioblastoma cells to RT across *in vitro* CRISPRi screens (Fig. 1a, b, Extended Data Fig. 2a, Supplementary Table 1) plus non-targeted control sgRNAs was delivered by CED, and mice were treated with either cranial RT or no RT and euthanized for tumor dissection 5 days after treatment. Dissociated single tumor cells expressing sgRNAs were isolated using FACS for mCherry and processed for single-cell RNA sequencing with direct sgRNA capture^32^, yielding a total of 16,114 single cell transcriptomes with sgRNA expression across 4 to 6 animals per condition (Extended Data Fig. 2b). Median on-target *in vivo* gene suppression was 96.8% and 95.8% in control and RT conditions, respectively (Extended Data Fig. 2c), and single glioblastoma cells exhibited heterogeneity across cell cycle, ribosome biogenesis, stemness, and cell stress pathways (Extended Data Fig. 2d, e).

sgRNA perturbation phenotypes were measured *in vivo* using differential gene expression and supervised gene set enrichment analyses, which showed significant alterations in 49 gene sets across 30 sgRNA perturbations that met established coverage and on-target knockdown criteria^32^ (Extended Data Fig. 3a, Supplementary Table 2). Both cell intrinsic (G2M targets, apoptosis) and cell extrinsic (interferon alpha response) pathways were dysregulated following CRISPRi perturbations, with some gene sets such as oxidative phosphorylation and epithelial mesenchymal transition showing divergent expression ± RT.

Supervised analyses using known gene sets can inform the interpretability of *in vivo* perturb-seq data, but to quantitatively model interactions between sgRNA perturbations and unbiased gene expression programs across single glioblastoma cells *in vivo*, GRN models were constructed using D-SPIN inference^33^ on perturb-seq data from GBM43 PDXs ± RT. Orthogonal nonnegative matrix factorization (oNMF) with Bayesian information criterion (BIC) identified 25 unbiased gene expression programs that optimally represented the effects of all 30 sgRNA perturbations *in vivo* (Fig. 1d, Extended Data Fig. 4a, b, Supplementary Table 3). These data-driven gene expression programs revealed both broad and granular molecular alterations that were not evident using supervised approaches (Extended Data Fig. 3a, Supplementary Table 2), including extracellular matrix remodeling (P16), the FOXM1 transcription network (P12), and interferon-mediated inflammation (P24).

To define the impact of sgRNA perturbations on glioblastoma cell responses to RT, linear discriminant analysis (LDA) uniform manifold approximation and projection (UMAP)^32^ was used to analyze single-cell transcriptomic heterogeneity. Three classes of *in vivo* perturbations were identified based on clustering of UMAP density metrics in the presence or absence of RT: (1) RT-independent distributed perturbations, which exhibited dispersed transcriptomic phenotypes either with or without RT, (2) RT-independent clustered perturbations, which exhibited organized transcriptomic phenotypes with or without RT, and (3) RT-dependent perturbations, like *PRKDC, RIF1,* and *CENPT*, which exhibited dispersed transcriptomic phenotypes without RT that became organized with RT (Fig. 1d, e, Extended Data Fig. 5a, b). We next mapped the interactions between perturbations and unbiased gene expression programs using D-SPIN according to RT-independent distributed, RT-independent clustered, or RT-dependent cluster assignments (Fig. 1f, Extended Data Fig. 6a). This demonstrated that activating and inhibitory interactions between perturbations and gene expression programs were distinct in the presence versus absence of RT, suggesting that RT-induced reprogramming of GRNs could be used to nominate targets for rational combination or sequential therapies to increase the efficacy of RT.

Thus, to nominate sgRNA perturbations that could be leveraged as therapeutic targets to sensitize glioblastomas to RT, we identified gene expression programs that exhibited significant activating or inhibitory interactions only in the presence of RT. This analysis revealed activating interactions between DNA repair (P10) and interferon-mediated inflammatory response (P24) after treatment with RT (Fig. 1g). We then asked whether interactions between sgRNA perturbations and gene expression programs could change polarity (*i.e*. sign flip) from inhibitory to activating when sgRNA perturbations occurred with RT. sgRNA suppression of *PRKDC* or *RIF1* activated interferon-mediated inflammatory response programs (P24) only in the presence of RT (Fig. 1d, f, g, Extended Data Fig. 6b), suggesting that *PRKDC* and *RIF1* may modulate both cell-intrinsic and tumor immune microenvironment signaling events from within malignant cells that are dependent on RT. Analysis of established gene sets from MSigDB confirmed enrichment of inflammatory signaling following sgRNA suppression of *PRKDC* that was amplified by RT (Extended Data Fig. 6c). Moreover, D-SPIN analysis of our published *in vitro* and intracranial *in vivo* perturb-seq data^32^ using the immunocompetent GL261 mouse syngeneic glioblastoma model validated activating interactions between inflammatory gene expression programs and *Prkdc* perturbation only in the presence of RT (Extended Data Fig. 7a-c). Given the multitude of RT-dependent gene expression changes following sgRNA suppression of *PRKDC*, and the existence of clinical small molecule inhibitors of DNA-PKcs^36,37,43,44^, we nominated *PRKDC* for functional and mechanistic interrogation as a pleotropic RT sensitizing target in glioblastoma.

## DNA-PKcs inhibition and radiotherapy reprogram malignant and immune microenvironment cells toward an inflammatory state in glioblastoma preclinical models

**Figure 2:**
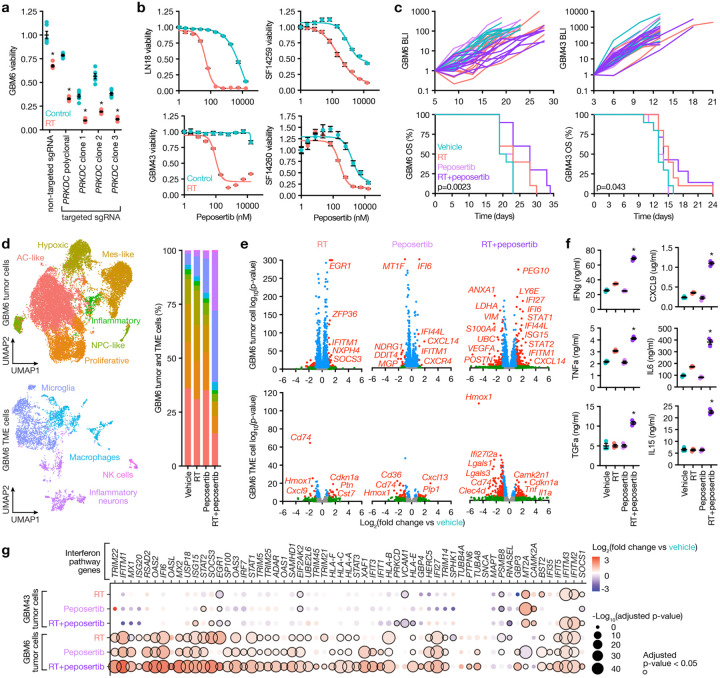
DNA-PKcs inhibition and radiotherapy reprogram malignant and immune microenvironment cells toward an inflammatory state in glioblastoma preclinical models. **a**, Viability of GBM6 cells with CRISPR/Cas9 deletion of *PRKDC* followed by polyclonal or monoclonal selection ± RT (2Gy × 2 daily fractions). Student’s t-tests, *p-value < 0.0001. **b**, Viability dose-response curves of glioblastoma cell cultures (LN18, GBM43) or glioblastoma primary tumorsphere cultures (SF14259, SF14260) after treatment with peposertib ± RT (2Gy × 2 daily fractions). Data are normalized to culture viability with 0 nM peposertib ± RT. **c**, Intracranial bioluminescent imaging (BLI, top) and Kaplan-Meier survival analyses (bottom, log-rank tests) of GBM6 (left) or GBM43 (right) PDXs (n=10 mice/arm) after treatment with RT (2Gy × 5 daily fractions), peposertib (100mg/kg × 5 daily treatments), or RT and peposertib. **d**, UMAPs of single-cell transcriptomes from intracranial GBM6 PDXs (n=8) treated with vehicle, peposertib (100mg/kg × 5 daily treatments), RT (2Gy × 5 daily fractions), or RT and peposertib showing malignant human cell states (top, n=18,456 cells) or tumor microenvironment (TME) mouse cell types (bottom, n=4,251 cells), corresponding to mouse cells of the immune-predominant cluster identified in Extended Data Fig. 8b. Stacked bar plot shows the proportion of malignant glioblastoma cell states and immune TME cell types across treatment conditions. **e**, Volcano plots showing differential expression analyses of single-cell RNA sequencing data from GBM6 malignant human cell states (top) or mouse TME cell types (bottom) across treatment conditions, each compared to vehicle control. Red, absolute value log_2_ fold change > 1 and adjusted p value < 0.00001. Blue, absolute value log_2_ fold change ≤ 1 and adjusted p value < 0.00001. Green, absolute value log_2_ fold change > 1 and adjusted p value ≥ 0.00001. Gray, absolute value log_2_ fold change ≤ 1 and adjusted p-value ≥ 0.00001. **f**, Multiplexed *in vitro* cytokine assays showing increased cytokine secretion into GBM6 culture supernatant 24 hours after treatment with RT (2Gy × 5 daily fractions), peposertib (300 nM), or RT and peposertib. ANOVA, *p-value < 0.001. **g**, Bubble plot of interferon pathway gene expression from single-cell RNA sequencing of intracranial GBM43 (top, n=16,009 cells) or GBM6 (bottom, n=18,456 cells) human malignant tumor cell states after treatment with RT (2 Gy × 5 daily fractions), peposertib (100 mg/kg × 5 daily treatments), or RT and peposertib. Lines represent means and error bars represent the standard error of the means.

To validate the RT sensitizing effects of *PRKDC/*DNA-PKcsinhibition in glioblastoma preclinical models, *PRKDC* was deleted from cultured GBM6 PDX cells using CRISPR/Cas9, which decreased *in vitro* cell viability compared to GBM6 PDX cells expressing non-targeted control sgRNAs (Fig. 2a). The combination of *PRKDC* deletion with RT resulted in enhanced loss of *in vitro* cell viability in polyclonal and monoclonal cultures after CRISPR/Cas9 editing (Fig. 2a). To validate these results pharmacologically, DNA-PKcs was inhibited across multiple glioblastoma cell cultures using peposertib, a small molecule ATP-competitive selective inhibitor of DNA-PKcs that is currently under investigation in multiple clinical trials for patients with advanced solid tumors, including glioblastoma (*e.g*. NCT04555577)^36–39^. The combination of peposertib with RT decreased cell viability in a drug dose-dependent manner when compared to peposertib alone in human glioblastoma cell lines (LN18), cultured glioblastoma PDX cells (GBM43), and patient-derived primary glioblastoma cell cultures grown as tumorspheres (SF14259, SF14260) (Fig. 2b).

To determine if pharmacological inhibition of DNA-PKcs sensitizes glioblastoma to RT *in vivo*, intracranial GBM6 or GBM43 PDXs were established in athymic mice that were treated with oral peposertib ± cranial RT. Combination therapy reduced intracranial tumor bioluminescent imaging (BLI) greater than vehicle-only controls (p<0.0001) or peposertib alone (p=0.0007) and trended toward significant reduction compared to RT alone (p=0.084, p=0.0147 across all arms, two-way ANOVAs) (Fig. 2c, left). Combination therapy also prolonged survival compared to vehicle (p=0.0019) or peposertib alone (p=0.003) and trended toward increased survival compared to RT alone (p=0.081, p=0.0023 across all arms, log-rank tests) (Fig. 2c, left).

To define tumor cell states and immune microenvironment cell types underlying glioblastoma responses to DNA-PKcs inhibition and RT *in vivo*, intracranial GBM6 PDXs were dissected 5 days after treatment and processed for single-cell RNA sequencing. Human GBM6 PDX cells (n=18,456) were distinguished from mouse TME cells (n=33,623) using species-specific mRNA alignment, which revealed 6 glioblastoma malignant cell states and 11 mouse TME cell types (Fig. 2d, Extended Data Fig. 8a-d, Supplementary Table 4). Iterative re-clustering of immune TME cells (n=4,251) showed macrophages, microglia, NK cells, and inflammatory neurons within the TME of GBM6 (Fig. 2d, Extended Data Fig. 8d). Comparison of tumor cell states and TME cell types across treatment conditions showed minimal changes between control and monotherapy treatments but significant expansion of immune TME cell types after combination therapy with peposertib and RT (Fig. 2d).

Pseudobulked differential expression analyses of single-cell RNA sequencing data from intracranial GBM6 PDXs showed enrichment of inflammatory and interferon genes (*CXCL14, IFITM1, STAT1, STAT2, IFI6, IFI27, ISG15*) and repression of tumor maintenance genes (*VEGFA, VIM*) in malignant cells after combination therapy with peposertib and RT compared to control (Fig. 2e, Supplementary Table 5). Similar analyses of pseudobulked immune TME cell types (macrophages, microglia, NK cells) showed enrichment of pro-inflammatory genes (*Il1a, Tnf*) after combination therapy with peposertib and RT (Fig. 2e). Multiplexed *in vitro* cytokine assays showed synergistic enrichment of pro-inflammatory cytokine secretion (IFNγ, CXCL9, TNFα, IL6, TGFα, IL15) into cultured GBM6 PDX cell supernatant after combination therapy with peposertib and RT compared to monotherapy treatments or control (Fig. 2f).

Combination therapy with peposertib and RT attenuated intracranial BLI and prolonged survival in mice with intracranial GBM43 PDXs as well, but efficacy was diminished compared to GBM6 PDXs (Fig. 2c, right). To test the hypothesis that differences in inflammatory gene expression after combination therapy may be associated with differences in anti-tumor efficacy across preclinical models, single-cell RNA sequencing was performed on GBM43 PDXs following treatment with peposertib and RT. Single-cell RNA sequencing revealed 6 malignant cell states (n=16,009 cells) and 11 TME cell types (n=10,192 all cells, 8,441 immune cells) in GBM43 PDXs (Extended Data Fig. 9a-f, Supplementary Table 4), but there were fewer differences in immune TME cell type composition across treatment conditions (Extended Data Fig. 9g) compared to TME changes in GBM6 (Fig. 2d). Differential expression analyses comparing malignant cells after treatment with RT, peposertib, or peposertib and RT showed greater enrichment of interferon pathway genes in GBM6 malignant cells than in GBM43 malignant cells (Fig. 2g). Differential activation of inflammatory gene expression (*Ifit1, Isg15, Isg20*) was also evident in TME cells of GBM6 compared to TME cells of GBM43 (Extended Data Fig. 9h), although to a lesser degree. These data suggest that reprogramming of both malignant cells and the immune TME underlies the efficacy of DNA-PKcs inhibition and RT across glioblastoma preclinical models.

## DNA-PKcs inhibition and radiotherapy reprogram malignant and immune microenvironment cells toward an inflammatory state in human glioblastomas

**Figure 3:**
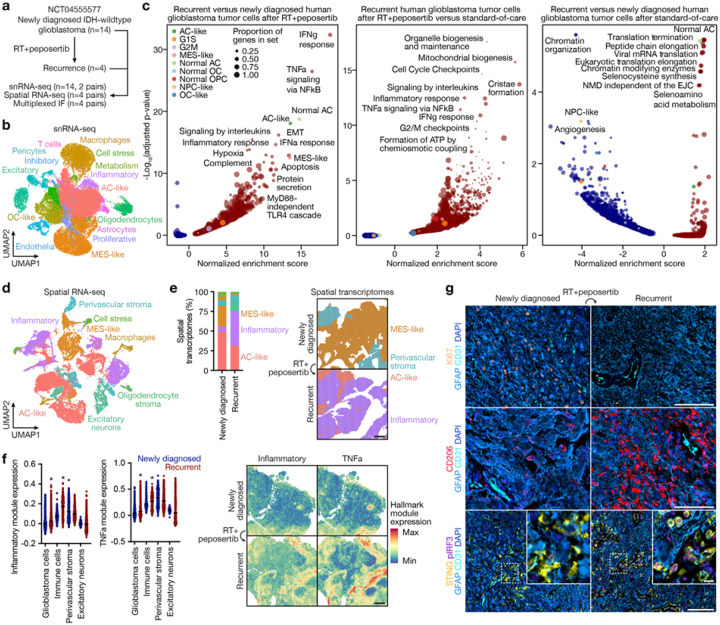
DNA-PKcs inhibition and radiotherapy reprogram malignant and immune microenvironment cells toward an inflammatory state in human glioblastomas. **a**, Schema for molecular analyses of newly diagnosed IDH-WT, MGMT promoter unmethylated, glioblastoma samples from a phase 1 clinical trial (NCT04555577) of RT (60Gy in 30 fractions) plus concurrent peposertib (300mg daily). **b**, UMAP analysis of 102,709 single-nucleus transcriptomes from 14 glioblastoma samples from patients who were treated with RT and peposertib on NCT04555577 (n=12 newly diagnosed, n=2 recurrent), 4 of which correspond to patient-matched pairs of newly diagnosed and recurrent samples from 2 patients. **c**, Volcano plots of gene set enrichments following differential expression analyses of single-nucleus RNA sequencing data comparing (1) recurrent glioblastoma malignant cells after RT and peposertib (n=2 samples, n=13,406 single-nuclei) versus newly diagnosed human glioblastoma malignant cells (n=12 samples, n=51,819 single-nuclei) from patients treated on NCT04555577 (left), (2) recurrent glioblastoma malignant cells after RT and peposertib versus recurrent glioblastoma malignant cells after standard-of-care treatment (n=46 samples, n=63,378 nuclei, middle), or (3) recurrent glioblastoma malignant cells after standard-of-care treatment versus newly diagnosed human glioblastoma malignant cells (n=40 samples, n=63,309 nuclei, right). **d**, UMAP analysis of 22,073 spatial transcriptomes from 4 patient-matched newly diagnosed (n=4) or recurrent (n=4) glioblastomas from patients treated with RT and peposertib on NCT04555577. **e**, Stacked bar plot showing the proportion of each spatial transcriptomic tumor cell state or microenvironment cell type across newly diagnosed or recurrent glioblastoma samples from NCT04555577. Representative spatial transcriptomic projections for a patient-matched pair demonstrating expansion of inflammatory glioblastoma tumor cells at the time of recurrence is shown on the right. Scale bar, 1mm. **f**, Distributions of inflammatory signaling or TNFα signaling modules across spatial transcriptomes in newly diagnosed or recurrent glioblastoma samples after RT and peposertib in glioblastoma malignant cells, immune cells, perivascular stroma, or excitatory neurons. Student’s t-tests, *p-value < 0.0001. Lines represent means and error bars represent the standard error of the means. Representative inflammatory response and TNFα signaling modules for a patient-matched pair of samples is shown on the right. Scale bar, 1mm. **g**, Multiplex sequential immunofluorescence for glioblastoma malignant cells (GFAP), nuclei (DAPI), proliferative cells (Ki67), endothelia (CD31), macrophages (CD206), STING, and the cGAS/STING effector pIRF3, showing decreased glioblastoma cell proliferation (top), macrophage enrichment (middle), and activation of cGAS/STING signaling (bottom) in patient-matched newly diagnosed and recurrent samples following RT and peposertib on NCT04555577. Scale bars: 100 μm or 10 μm (inset).

To determine if DNA-PKcs inhibition and RT also lead to reprogramming of malignant cells and the immune TME to an inflammatory state in human glioblastoma, single-nucleus RNA sequencing, spatial RNA sequencing, and multiplexed sequential immunofluorescence were performed on patient-matched newly diagnosed and recurrent IDH-WT MGMT promoter unmethylated glioblastoma samples from a phase 1 clinical trial of postoperative RT plus concurrent peposertib (NCT04555577) (Fig. 3a). Single-nucleus RNA sequencing of 14 fresh frozen glioblastoma samples (n=12 newly diagnosed, n=2 recurrent) revealed 7 malignant cell states and 8 TME cell types across 102,709 single-nucleus transcriptomes that were defined using inferred copy number variants and marker gene expression (Fig. 3b, Extended Data Fig. 10a-c, Supplementary Table 6). Differential expression analyses in malignant cells followed by gene set enrichment analyses revealed that IFNγ, IFNα, and TNFα gene expression programs were significantly enriched after combination therapy with peposertib and RT in recurrent compared to newly diagnosed human glioblastomas from patients treated on NCT04555577 (Fig. 3c, left, Supplementary Table 7). In support of these data, recurrent glioblastoma malignant cells after combination therapy with peposertib and RT showed significant enrichment of inflammatory response, TNFα, and IFNγ gene expression programs when compared to recurrent glioblastoma malignant cells after standard-of-care treatment from a published cohort of 46 recurrent glioblastoma samples comprising 63,378 single-nucleus transcriptomes^11^ (Fig. 3c, middle). Reanalysis of single-nucleus RNA sequencing data from the 46 recurrent and 40 newly diagnosed glioblastoma samples in this published cohort, including 36 patient-matched pairs (n=63,309 nuclei)^11^, did not show enrichment in inflammatory signaling pathways in recurrent compared to newly diagnosed malignant cells after standard-of-care treatment (Fig. 3c, right). These data suggest that reprogramming of glioblastoma tumors toward an inflammatory state in preclinical models in the short-term (Fig. 2) could persist long-term after conclusion of treatment in human tumors.

To determine if inflammatory reprogramming after DNA-PKcs inhibition and RT was conserved within individual patients, spatial RNA sequencing was performed on formalin-fixed paraffin-embedded (FFPE) sections from 4 patient-matched newly diagnosed (n=4) and recurrent (n=4) glioblastoma samples after treatment with peposertib and RT on NCT04555577. UMAP analysis of 22,073 spatial transcriptomes meeting quality control criteria showed 4 glioblastoma malignant cell states and 4 TME cell types, which were defined using inferred spatial copy number variants and marker gene expression (Fig. 3d, Extended Data Fig. 11a, Supplementary Table 8). Comparison of recurrent to newly diagnosed glioblastoma spatial transcriptomes revealed expansion of inflammatory glioblastoma cells and contraction of AC-like and MES-like glioblastoma cells after combination therapy with peposertib and RT (Fig. 3e, Extended Data Fig. 12a). Inflammatory response and TNFα expression modules were enriched in recurrent compared to newly diagnosed malignant and immune TME spatial transcriptomes, but not in neuronal spatial transcriptomes (Fig. 3f, Extended Data Fig. 12b). Inflammatory expression modules were elevated in the perivascular stroma in both newly diagnosed and recurrent glioblastoma samples (Fig. 3f), and analysis of gene expression along spatial trajectories revealed that inflammatory, interferon, and TNFα expression modules were elevated immediately adjacent to the perivascular stroma but decreased to baseline levels within ~2 mm in tumor tissue (Extended Data Fig. 12c). These data suggest that peposertib promotes heterogeneous intratumor inflammatory signaling that is enriched in the perivascular space.

To validate inflammatory reprogramming and shed light on mechanisms of response to DNA-PKcs inhibition and RT, multiplexed sequential immunofluorescence was performed on FFPE sections from 4 patient-matched newly diagnosed (n=4) and recurrent (n=4) glioblastoma samples after treatment with peposertib and RT on NCT04555577. Recurrent compared to patient-matched newly diagnosed glioblastoma samples demonstrated reduced Ki67 and increased CD206+ myeloid cells (Fig. 3g), which are tumor associated macrophages that can induce anti-tumor immunity^45,46^. TME cells in recurrent glioblastoma samples after treatment with peposertib and RT also showed increased pIRF3/STING co-expression, consistent with cGAS/STING-mediated activation of interferon and inflammatory gene expression programs that were revealed by orthogonal assays in human samples (Fig. 3c, f) and preclinical models (Fig. 2e–g).

## cGAS/STING activation potentiates response to DNA-PKcs inhibition and radiotherapy in glioblastoma preclinical models

**Figure 4:**
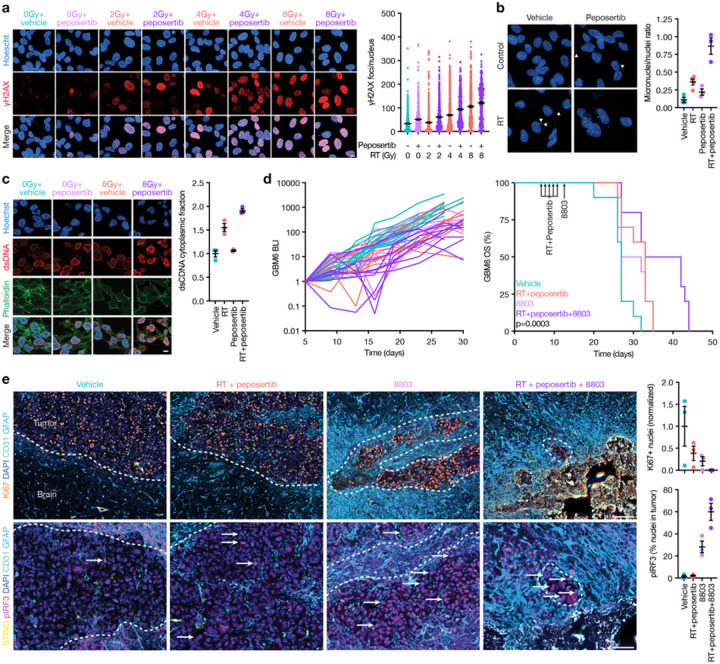
cGAS/STING activation potentiates response to DNA-PKcs inhibition and radiotherapy in glioblastoma preclinical models. **a**, Immunofluorescence for γH2AX and nuclei (Hoescht) demonstrating increasing double strand DNA breaks in GBM6 cells after treatment with peposertib (400nM), RT (2, 4, or 8 Gy), or RT and peposertib. Scale bar, 10μm. Quantification of γH2AX foci per nucleus (n=358–771 nuclei per condition) is shown on the right. ANOVA, p-value < 0.0001. **b**, Confocal micrographs of GBM6 cell nuclei (Hoescht) after treatment with RT (8Gy × 1 fraction), peposertib (400nM), or RT and peposertib. Arrowheads show micronuclei. Scale bar, 10μm. Quantification of micronuclei across 3–4 biological replicates per condition is shown on the right. ANOVA, p-value < 0.0001. **c**, Immunofluorescence for dsDNA (n=602–884 cells per condition), phalloidin, and nuclei (Hoescht) demonstrate accumulation of cytoplasmic dsDNA in GBM6 cells following treatment as in **b**. Scale bar, 10μm. dsDNA quantification following cytoplasmic biochemical fractionation across 3 biological replicates per condition is shown on the right. ANOVA, p-value < 0.0001. **d**, Intracranial bioluminescent imaging (left, BLI) and Kaplan-Meier survival analyses (right, log-rank tests) of GBM6 PDXs (n=10 mice/arm) after treatment with RT (2Gy × 5 daily fractions) and peposertib (100mg/kg × 5 daily treatments) on days 5–9, STING agonist 8803 (2.5 μg by CED on day 12), or RT and peposertib and 8803. **e**, Multiplex sequential immunofluorescence and quantification for Ki67 and pIRF3 in intracranial GBM6 2 days following the conclusion of treatment. Architectural markers for tissue orientation and cellular identification include endothelia (CD31), astrocytes (GFAP), and nuclei (DAPI). Regions of interest denoted by the dashed white lines included the highly proliferative tumor cells. Arrows show representative pIRF3+ cells. Images are representative of 3 biological replicates per condition. Scale bars, 50μm (top), 25μm (bottom). ANOVA, p-value < 0.001. Lines represent means and error bars represent the standard error of the means.

In support of the connections between DNA-PKcs inhibition, RT, and cGAS/STING activation in human glioblastomas, combination therapy of cultured GBM6 PDX cells or human glioblastoma cell lines (LN18) with peposertib and RT resulted in enrichment of DNA double strand breaks (Fig. 4a), micronuclei (Fig. 4b), and cytoplasmic double strand DNA (Fig. 4c) compared to monotherapy or control treatment conditions. These data validate that DNA-PKcs inhibition and RT enhance genomic stress and DNA damage when given in combination to glioblastoma. cGAS/STING is activated by cytoplasmic DNA, and considering the enrichment of pro-inflammatory signaling in malignant tumor cells as well as the TME following DNA-PKcs inhibition and RT in preclinical glioblastoma models (Fig. 2) and human glioblastomas (Fig. 3), we hypothesized that further intratumor immune modulation using a cGAS/STING agonist could increase the anti-tumor efficacy of DNA-PKcs inhibition and RT. To test this, intracranial GBM6 glioblastoma PDXs were treated with oral peposertib and cranial RT ± CED of 8803, a cyclic dinucleotide STING agonist with efficacy in glioblastoma preclinical models^47–49^. Triple therapy reduced intracranial tumor BLI greater than control (p=0.0113, two-way ANOVA) and extended survival compared to control, 8803 alone, and peposertib + RT (p=0.0003, log-rank test) (Fig. 4d). Histological analysis confirmed that intracranial tumors were smaller after triple therapy and exhibited increased necrosis and hemorrhage compared to all other treatment conditions (Extended Data Fig. 13a, b). Multiplexed sequential immunofluorescence demonstrated reduced Ki67+ tumor cells and increased pIRF3+ cells 2 days following the end of triple therapy, consistent with anti-tumor efficacy and downstream STING activation in response to this new therapeutic approach (Fig. 4e).

Here we integrate genome-wide *in vitro* and single-cell *in vivo* functional genomics in preclinical models with single-nucleus and spatial transcriptomic sequencing of human samples to identify genetic vulnerabilities that sensitize glioblastoma to RT. Using GRN modeling, we identify *PRKDC*, the catalytic subunit of DNA-PK, as a clinically actionable treatment sensitizing target that influences tumor cells and the TME in glioblastoma. Genetic suppression or pharmacologic inhibition of DNA-PKcs using a clinically available small molecule increases the anti-tumor efficacy of RT across a preclinical models. Analyses of glioblastoma tumor samples from preclinical models and a phase 1 clinical trial (NCT04555577) reveals that DNA-PKcs inhibition with concurrent RT reprograms both tumor and TME cells toward pro-inflammatory states through immunostimulatory genomic stress. Finally, we show that anti-tumor immune priming in response to DNA-PKcs inhibition and RT can be amplified using a clinically-available STING agonist. These data establish a technical framework for identifying response or resistance mechanisms to cancer treatment, elucidate fundamental biology underlying the most common malignant brain tumor in adults, and establish rationale for combination and sequential therapies that target the DNA damage response and the immune microenvironment to enhance the efficacy of RT, an emerging therapeutic strategy being explored in other cancers^50,51^.

These results are supported by prior studies identifying DNA-PKcs as a key regulator of DNA damage repair and a determinant of therapeutic resistance in advanced cancers, including glioblastoma^34,35,52,53^. DNA-PKcs regulates NHEJ, a key pathway for repair of DNA double strand breaks that can be induced by ionizing radiation^34,35^, and synergistic induction of DNA double strand breaks in tumor cells is a promising therapeutic approach in malignant gliomas^54–58^. Although DNA-PKcs has been well described in NHEJ and V(D)J recombination in lymphocytes^59,60^, the mechanisms underlying DNA-PKcs-mediated treatment resistance in glioblastoma were incompletely understood. Our data demonstrate that non-canonical signaling through the TME contributes to DNA-PKcs-mediated responses to RT, a biological finding that links the DNA damage response to the immune microenvironment with direct relevance to patients^36^. Other non-canonical functions of DNA-PKcs may also be at play, including DNA-PKcs regulation of glioma stem cells through post-translational modifications that are independent of NHEJ^61^. Beyond glioblastoma, DNA-PKcs has been shown to directly regulate transcription^62^, post-translational modification^63^, metabolism^64,65^, and immunity^66,67^. How or even if these diverse mechanisms of DNA-PKcs activity may contribute to treatment resistance in glioblastoma is incompletely understood.

DNA-PKcs inhibition has been shown to enhance RT-induced cytotoxicity and increase immune signaling, including through direct and indirect activation of cGAS/STING^66–68^. In antiviral innate immunity, DNA-PKcs deficiency leads to cGAS/STING activation through accumulation of cytosolic DNA^66^, a finding that we also observed in glioblastoma cells. Recent studies have shown that direct activation of the cGAS/STING pathway enhances anti-tumor immunity, leading to therapeutic efficacy in preclinical studies^47–49,69–73^ and the initiation of clinical trials in glioma and other malignancies^74–76^. Our recent observation that cGAS/STING agonism can induce opening of the blood-brain barrier and potentiate the anti-tumor efficacy of RT enables new combination treatment strategies that leverage drugs with otherwise poor penetrance for glioblastoma, including other inhibitors of the DNA damage response^47,77^. Our results provide an unbiased, functional genomic-driven rationale for combining DNA-PKcs inhibition with RT and cGAS/STING agonism, demonstrating that DNA-PKcs inhibition and RT primes glioblastoma for inflammatory signaling that can be further enhanced by cGAS/STING activation. Analogous strategies combining DNA damage response therapies with systemic immunotherapies are areas of active investigation in other cancers^78–80^.

Our study provides a framework for integrative analyses of functional and clinical genomics data to identify actionable therapeutic targets that could be applied to other cancers or non-cancerous diseases in patients. However, some limitations must be considered, including the limited number of targets assessed by *in vivo* perturb-seq, which restricted target discovery to a panel of 35 genes nominated from genome-wide *in vitro* CRISPRi screens. Our analyses of human glioblastoma samples treated with DNA-PKcs inhibition and RT provides direct clinical relevance and external validity for our findings in preclinical models, but these phase 1 clinical trial samples were not obtained at a window-of-opportunity prior to clinical tumor recurrence. This limits our ability to directly attribute molecular phenotypes to the treatments patients received. Nevertheless, our data demonstrate how integrated genomics can uncover convergent genetic and therapeutic vulnerabilities in glioblastoma, leading to mechanistic rationalization for the design of combination or sequential therapies. These findings support further clinical investigation of the interplay between regulators of the DNA damage response and immune modulation as a strategy to overcome treatment resistance in patients living with glioblastoma.

## Methods

### Ethics and institutional approval

This study complied with all relevant ethical regulations and was approved by the University of California San Francisco Institutional Review Board (15–17500), the University of California San Francisco Institutional Animal Care and Use Committee (AN204686), and the MD Anderson Cancer Center Institutional Review Board (2019–1035). As part of routine clinical practice, all patients included in this study signed a written waiver of informed consent to contribute de-identified tissue for research.

### Cell culture

HEK-293T, GL261, SB28, and LN18 cells were cultured in Dulbecco’s Modified Eagle Medium (Gibco, #11960069) supplemented with 10% fetal bovine serum (Life Technologies, #16141), and antibiotic-antimycotic (Thermo Scientific, #15240062). Cell cultures were authenticated by STR analysis at the UC Berkeley DNA Sequencing Facility and were routinely tested for mycoplasma using the MycoAlert Detection Kit (Lonza, #75866–212). Glioblastoma cell lines and patient-derived xenografts (PDX) stably expressing the CRISPR interference (CRISPRi) machinery dCas9-Zim3 were generated as previously described^32^. Lentivirus was produced by transfecting HEK293T cells with packaging vectors (pMD2.G from Addgene and pCMV-dR8.91 from Trono Lab) following the manufacturers protocol (Mirus, #MIR6605). Primary glioblastoma cell cultures (S14259, SF14260) were derived from patient samples that were collected from the operating room at the University of California San Francisco as previously described^31^. Primary glioblastoma cell cultures were grown in Neurobasal-A Medium (Gibco, #10888022) supplemented with B-27 (Gibco, #12587010), N-2 (Gibco, #17502–048), plasmocin prophylactic (Invivogen, #ant-mpp), primocin (Invivogen, #ant-pm-1), EGF 20ng/mL (VWR, #10781–694), and FGF 20ng/mL (PeproTech, #10777–988).

### In vitro CRISPRi screens

CRISPRi screens were performed as described previously^25,31^. Glioblastoma cell lines stably expressing CRISPRi machinery were transduced with lentivirus supernatant containing the dual sgRNA human V3 CRISPRi library^40^, which targets 20,528 human genes and encodes 1025 non-targeted control sgRNAs (sgNTC), for LN18 cells; or the V2 mouse CRISPRi library^41^, which encodes 107,415 sgRNAs targeting 20,003 mouse genes along with 2,170 non-targeted control sgRNAs, for SB28 cells. Screens were performed in triplicate (LN18) or duplicate (SB28) cultures with coverage of at least 500x cells per target gene. Cells expressing sgRNAs were selected using puromycin 1μm/mL for 48hr and transferred to puromycin-free growth media for 24hr. Initial (T0) cell populations were frozen in 10% DMSO and processed for genomic DNA alongside experimental samples at endpoints that corresponded to 10 population doublings in control (e.g. un-irradiated) conditions. Replicate screens were performed in the presence of fractionated radiotherapy (RT, 2Gy × 2) delivered daily following the T0 timepoint using the X-RAD 320 kV cabinet irradiator (Precision X-Ray). Genomic DNA was harvested using the NucleoSpin Blood L (LN18) or XL (SB28) Kits (Machery-Nagel) for each cell population, and sgRNA cassettes were amplified using 22 cycles of PCR and NEBNext Ultra II Q5 PCR MasterMix (New England Biolabs, #M0544L). Sequencing was performed on a NovaSeqX (Illumina) using custom sequencing primers^40,41^, and analysis was performed using ScreenPro2 (https://github.com/ArcInstitute/ScreenPro2). GL261 mouse CRISPRi screen data, corresponding to 27,300 sgRNAs targeting 5,234 cancer related and/or druggable mouse genes, in addition to 530 non-targeted control sgRNAs, were obtained from our prior report^32^ and analyzed in parallel. sgRNAs with fewer than 50 mean read alignments at T0 were omitted from analysis, and pseudocount of 0.5 was added to counts of 0. Growth phenotype was defined as log_2_(sgRNA count Tend / sgRNA count T0). RT/vehicle ratio was defined as log_2_(sgRNA count Tend (RT) / sgRNA count Tend (vehicle)). Statistical significance was defined using a two-sided Student’s t-test comparing replicate distributions of library-normalized counts for each sgRNA between conditions or time points in the case of the V3 library, and Mann-Whitney U test in the case of the V2 library. Hit calling was performed by calculating a combined score that was defined as the product of z-standardized phenotype × negative log_10_(p-value), and setting a threshold that corresponded to an empirical false discovery rate of 1–2% for non-targeted control hits, which corresponded to combined scores of 8 for LN18, 10 for SB28, and 8 for GL261.

### Intracerebral tumor establishment and treatments

Firefly luciferase-expressing GBM43 or GBM6 PDX cells with or without stable expression of CRISPRi machinery were intracerebrally injected into the right caudate putamen of female athymic (nu/nu, homozygous), 5- to 6-week-old mice from Envigo, as previously described^81^. Each mouse was injected with 300,000 tumor cells in 3μL suspension into the right caudate putamen at a rate of 1μL/min. Mice were treated with 100mg/kg of peposertib by oral gavage and/or cranial RT (2Gy per fraction) starting at 5 days after tumor cell implantation, as described below. STING agonist 8803 was delivered as a single dose by convection enhanced delivery (CED) 3 days following the final fraction of RT, as described below.

### Bioluminescence imaging of intracranial tumor growth

Intracranial tumor size was measured using bioluminescent imaging by intraperitoneal injection of 150mg/kg luciferin (D-luciferin potassium salt, Gold Biotechnology) on an IVIS Lumina imaging station equipped with Living Image software (Caliper Life Sciences).

### Convection enhanced delivery of sgRNA lentivirus libraries or STING agonist

CED infusion cannulas were constructed with silica tubing (Polymicro Technologies) fused to a 0.1ml syringe (Plastic One) with a 0.5-mm stepped tip needle that protruded from the silica guide base. The syringe was loaded with 15μL concentrated lentivirus produced using the LV-MAX Lentiviral Production System (Thermo Fisher #A35684), followed by 200x concentration using ultracentrifuge (Beckman L8–80M) at 25,000g for 2.5hrs at 4°C. STING agonist IACS-8803 diammonium (MCE, # HY-130115B) was prepared as a single administration of 2.5μg in 10μL solution and delivered 3 days following the final fraction of RT. Mice were anesthetized with a combination of intraperitoneal injection of a mixture containing ketamine (100mg/kg) and xylazine (10mg/kg), and inhalation of isoflurane. The puncture hole used for intracranial tumor establishment (described above) was identified at the surface of the skull 3.0mm to the right of the bregma and immediately rostral of the coronal suture. A Hamilton syringe was attached to a microinfusion pump (Bioanalytical Systems), and the syringe with silica cannula was lowered through the puncture hole made in the skull, targeting the same region in the caudate putamen where tumor cells had been previously implanted. The payload was infused at a rate of 1μL/min until a volume of 10–15μL had been delivered. Cannulas were removed 2 minutes after completion of infusion. The skull surface was then swabbed with hydrogen peroxide before the hole was sealed with bone wax to prevent reflux, and the scalp was closed with surgical staples.

### Cranial irradiation

Mice were anesthetized with a combination of intraperitoneal injection of a mixture containing ketamine (100mg/kg) and xylazine (10mg/kg), and inhalation of 2.5% isoflurane with 1L of oxygen per minute for 5 minutes prior to positioning on an irradiation platform located 16.3cm from a Cesium-137 source (J. L. Shepherd & Associates). Subjects’ eyes, respiratory tracts, and bodies were protected with lead shielding. Cranial irradiation (2Gy × 2–5 daily fractions) was delivered at a dose rate of 247cGy/min. After irradiation, mice were monitored until recovery from anesthesia. Following no radiation or radiation treatments, mice were euthanized, brains were quickly removed from the skull, and tumors were micro-dissected.

### In vivo perturb-seq

The top two scoring sgRNAs for each of 35 target genes nominated based on depletion phenotypes from *in vitro* genome-wide CRISPRi screens described above, as well as two non-targeted control sgRNAs, were cloned into dual sgRNA lentivirus expression vectors with direct capture tags (Addgene, #187241) using NEBuilder HiFi DNA Assembly Master Mix (New England Biolabs, #E2621L). Concentrated lentivirus encoding pooled sgRNA libraries was produced using the LV-MAX Lentiviral Production System (Thermo Fisher, #A35684). GBM43 PDX cells expressing luciferase and CRISPRi machinery were intracranially implanted into mice as described above. Tumors were permitted to establish and expand for 5 days, and CED of concentrated mCherry-tagged lentivirus was performed as described above. For the radiation treatment arm, cranial irradiation was delivered to a dose of 2Gy × 2 daily fractions as described above, starting 2 days after CED to allow for lentiviral transduction and sgRNA expression. For subjects in the no radiation arm, tumor harvest was performed 5 days following CED. For subjects in the RT arm, tumor harvest was performed 2 days following completion of RT. Harvested tumors were minced and dissociated to single cell suspension using the Papain Dissociation System (Worthington, #LK003150) without the use of ovomucoid protease inhibitor. Cell suspensions were passed through a 70μm strainer (Corning, #352350), centrifuged at 300g for 5 minutes, and resuspended in cold phosphate buffered saline. sgRNA-expressing cells were isolated using FACS for mCherry, and sorted cells were processed for single-cell RNA sequencing with direct capture of sgRNA tags using a 10x Chromium Controller (10x Genomics, #1000204). 2 tumors from biological replicate animals in each arm were pooled to increase sgRNA-expressing cell recover, yielding 4–6 pooled replicates per treatment condition. Single-cell perturb-seq libraries were processed using the Chromium Next GEM Single Cell 3’ GEM, Library & Gel Bead Kit v3.1 with Feature Barcoding (10x Genomics, #1000269), allowing direct capture of modified sgRNAs, and sequenced on a NovaSeq-6000 (Illumina).

### Computational analysis of in vivo perturb-seq

Preprocessing: Library demultiplexing, alignment to a combined GRCh38 and mm10 reference, and UMI quantification was performed in CellRanger (10x Genomics). Subsequent analyses took place within the Seurat framework (v5.3), where cells with fewer than 200 features were removed and tumor cells were identified based on marker gene expression following Leiden clustering.

Differential gene expression analysis: Differential expression analysis was performed using DElegate (v1.1.0) as previously described^32,82^. In brief, DElegate wraps DESeq2, and adapts bulk sequencing analysis methods for single-cell data. Wald-based differential expression tests were performed on randomly assigned 3-group pseudo-replicates between cells with different treatments, cell states, or group designations.

Gene set enrichment analysis (GSEA): GSEA inputs were DElegate outputs, as described above. First, genes that did not pass independent filtering through DESeq2 were removed. Next, genes were ranked by log fold changes, and GSEA was performed using fgsea (v1.27.1) and msigdbr (v7.5.1). The maximum size of allowed gene sets was 500 and the eps parameter was 0 to allow for arbitrarily low p-values. Bubble plots were generated to show normalized enrichment scores for all pathways with at least one significant enrichment, defined as having an adjusted p-value < 0.05.

Linear discriminant analysis (LDA): To separate sgRNA perturbations in transcriptomic space, LDA was performed on all perturbation conditions ± RT. First, low coverage perturbations (≤ 5 cells) or those without evidence of on-target knockdown (*OVCH1, GRAMD4, FBXL16, NUP205, CDCA5*) were filtered. The CalcPerturbSig function in Seurat was used to generate perturbation signatures based on 50 principal components and 30 nearest neighbors. Cells were separated by treatment condition and processed using the MixscapeLDA function in Seurat with a log_2_ fold change threshold of 0.1 and 10 principal components. For each perturbation ± RT condition, cell density was visualized in uniform manifold approximation and projection (UMAP) space using the do_NebulosaPlot function in SCPubr (v 2.0.0). Density calculations were performed for each perturbation ± RT combination, where UMAP1/UMAP2 coordinates were divided into 10,000 evenly spaced rectangular areas and gaussian kernel densities were calculated for each. After min-max normalizing each density grid, 5 features were calculated for each perturbation density pattern: the mean density under ± RT conditions, the number of grid portions with density greater than the 50^th^ percentile density under ± RT conditions, which we term area, and the difference in area between RT and no RT treatments for the same perturbations. After standardizing each feature, the resulting feature vectors were hierarchically clustered with average linkage and the resulting dendrogram was pruned to three clusters. Based on these three clusters, perturbations were grouped into RT-independent distributed, RT-independent clustered, and RT-dependent classes.

### Dimension-scalable single-cell perturbation integration network (D-SPIN)

The D-SPIN framework constructs interpretable gene regulatory networks (GRNs)^33^ from perturb-seq data. These networks take the form of a graph ***J***, where nodes are gene expression programs and edges between nodes represent interactions between gene expression programs. D-SPIN models each sgRNA perturbation as a node fully connected to all gene program nodes, and perturbation-gene program edges represent how a perturbation modifies gene program expression. Thus, a response vector ***h*** is also described for each perturbation. The values of ***J*** and ***h*** are learned via a physics-inspired spin network model, details for which have been previously reported^33^. D-SPIN was used to construct a GRN of GBM43 *in vivo* perturb-seq data ± RT. Each of the steps of D-SPIN are described separately below: (1) preprocessing of single-cell RNA sequencing data, (2) selection of the number of gene expression programs for orthogonal non-negative matrix factorization (oNMF), (3) running oNMF and interpreting the resulting gene expression programs, (4) learning ***h*** for each perturbation and ***J*** by fitting the spin model, and (5) computational validation of the learned parameters.

D-SPIN preprocessing:CellRanger was used for GRCh38 alignment and sgRNA assignment and quantification. D-SPIN (v1.9.8) is implemented in Python, so single-cell preprocessing was performed with Scanpy (v2.0.0)^83^ using cells with at least 100 detectable features and features found in at least 3 cells. All cells were normalized to have a total count equal to the median of total counts before normalization and a log pseudocount 1 transformation was applied. Considering physiological differences between RT and no RT treatment conditions, the top 2000 highly variable genes were identified using cells from treatment conditions separately and the union of these genes was used for highly variable gene set analyses. The first 50 principal components were calculated and used for UMAP visualization, and cells were clustered using the Leiden algorithm with a resolution of 1.0. Cluster marker genes were identified using a Wilcoxon rank sum test.

Selection of oNMF parameters: D-SPIN uses oNMF to perform unsupervised discovery of gene expression programs. As with other non-negative matrix factorization methods, oNMF requires rank specification, which determines the number of gene expression programs. The D-SPIN framework uses two methods to choose a rank that captures the most variation without overfitting, one relying on the Bayesian Information Criterion (BIC) and one using the elbow method on a denoised expression matrix. For the first, 10 iterations of oNMF for range (5, 100) in increments of 5 were performed, calculating the BIC at each hyperparameter value. For the second, Markov affinity-based graph imputation of cells (MAGIC v3.0.0) was used to denoise the expression matrix, and the number of genes that significantly correlated with the resulting gene expression programs were compared to background.

oNMF and interpretation of gene programs: oNMF was computed on the pooled highly variable feature expression matrix described above (18,938 cells × 1812 highly variable genes that passed D-SPIN expression filters), repeating each computation 20 times and averaging the results via the built-in gene expression program discovery function in D-SPIN. The resulting gene expression programs were annotated based on the first 100 gene members of each using literature review, Enrichr outputs, and large language (LLM) annotation.

Network inference and visualization: After determining gene expression programs, cells that received sgRNAs targeting *OVCH1, GRAMD4,* or *FBXL16* were removed due to insufficient on-target knockdown. Cells that received sgRNAs targeting *NUP205* or *CDCA5* were removed due to insufficient cellular coverage. The remaining cells were divided into RT and no RT conditions, inferring a separate GRN for each treatment condition using the D-SPIN pseudo-likelihood method and a learning rate of 0.05 across 200 epochs. Normalized perturbation response vectors **h** were calculated for each sgRNA perturbation, with normalization by the cells that received non-targeted sgRNAs and no RT. For GRN network plots, only ***J*** and ***h*** edges with a weight less than −0.1 or greater than 0.1 and edges ranging [−1, 1] were visualized. For example, RT-specific gene expression program edges refer to edges that have an absolute value weight greater than 0.1 under RT conditions, but not under no RT conditions. Perturbations were grouped into separate plots based on assignment to RT-independent clustered, RT-independent distributed, and RT-dependent groups. Each GRN was visualized in front of single-cell UMAP embeddings, with nodes (P_n_) placed at the centroid of cells expressing the corresponding gene programs at the 99.5^th^ percentile, accounting for some repulsion to prevent overlap (using the assign_program_position function in D-SPIN). Perturbations were grouped along the arc based on simultaneous optimization over positive and negative edges to gene expression programs using Reichardt and Bornholdt’s Potts model, as implemented in the RBConfigurationVertexPartition module (v0.10.2) in the leidenalg package. For the sign flip edge interaction matrix, all edges were visualized.

Computational validation of the model via uncertainty analysis: To understand the confidence that could be assigned to our network inference, D-SPIN optimization has a unique solution, but suboptimal solutions may have similar loss function values. Thus, the optimization landscape around our parameters was calculated using Fisher information matrices. Specifically, a Fisher information matrix was calculated for GRNs inferred under both RT and no RT networks using the Schur complement of the parameter matrix. As the inverse of the Fisher information matrix is the Cramer-Rao bound, we used the matrix to quantify the variance in parameter estimation for each edge. In the vast majority of cases, the variance was an order of magnitude smaller than the magnitude of the parameters. Additionally, the posterior distribution of inferred network parameters should converge to a normal distribution centered on the inference results, with covariance given by the inverse of the Fisher information matrix. Therefore, 100,000 network solutions were sampled using the Fisher information matrix as the covariance matrix and our inferred ***J*** as mean. For each alternative network, the proportion of edges matched edges in ***J*** in one of two ways: either both inferred and sampled edges did not record strong interactions between programs, or both inferred and sampled edges exhibited strong interactions of the same sign.

Reanalysis of GL261 perturb-seq data: Preprocessing, oNMF, and D-SPIN network construction using *in vitro*, pre-infected *in vivo*, and CED *in vivo* perturb-seq data as previously described^32^, were performed as described above. BIC identified 15 gene expression programs after CED of sgRNAs compared to 25 gene expression programs in pre-infected and *in vitro* perturb-seq datasets. All sgRNA perturbations passing prior coverage thresholds were included for D-SPIN networks, but only *Prkdc* was isolated for visualization.

### CRISPR/Cas9 deletion of PRKDC

A pool of 3 sgRNAs targeting *PRKDC* was obtained from the Synthego GKOv2 kit. RNP’s were assembled using a 9:1 sgRNA to Cas9 ratio and electroporated into GBM6 cells using the DS-126 program on the Lonza 4D Nucleofector. Cas9 deletion was confirmed using Sanger sequencing of polyclonal and clonal populations.

### Drug dose-response viability and survival assays

Glioblastoma cells were seeded at 1,000–5,000 cells/well across 6 technical replicates per dose on a 96-well plate. Peposertib (Selleckchem, #S8586) was added to cell suspensions by serial dilution followed by RT on the X-RAD 320 kV cabinet irradiator (Precision X-Ray). Cell Titer Glo was performed using the manufacturer’s recommendations (Promega, #G7571) 7 days after drug exposure. Luminescence was measured using a GloMax 96 Microplate Luminometer (Promega, #E6521).

### *In vitro* cytokine profiling assays

GBM6 cells were seeded onto 6-well plates coated with 1% Geltrex (ThermoFisher, #A1413302) at a density of 400,000 cells per well. After seeding, drug treatment wells received peposertib dissolved in DMSO to an in-well concentration of 300nM. Control wells received the same volume of DMSO, and the total amount of DMSO did not exceed 2% by volume for any condition. After cell attachment, radiation condition plates received 5 fractions of 2Gy each, spaced 24 hours apart on the X-RAD 320 kV cabinet irradiator (Precision X-Ray). 24 hours after the 5^th^ fraction of radiation, 600μL of supernatant was centrifuged to pellet debris, and the top 300μL was isolated from each condition for submission to Eve Technologies for Human Cytokine/Chemokine Panel A 48-plex Discovery Assay (HD48A). Each control or treatment condition was analyzed in triplicate.

### Single-cell RNA sequencing of patient-derived glioblastoma xenografts

Tumors were dissociated as described above and processed for single-cell RNA sequencing using the 10x Chromium Controller (10x Genomics, #1000204). Single-cell RNA sequencing libraries were generated using the Chromium Next GEM Single Cell 3’ GEM, Library & Gel Bead Kit v3.1 and sequenced on a NovaSeq-6000 (IIllumina). Library demultiplexing, alignment to a combined GRCh38 and mm10 reference, and UMI quantification was performed in CellRanger (10x Genomics). For each sample, ambient mRNA was removed using SoupX^84^ and filtering was performed for features detected in at least 3 cells and cells with more than 200 features overall. Cells were defined as human or mouse (e.g. tumor or TME, respectively) if 90% of detected features were human or mouse. After pooling samples, cells with fewer than 15% mitochondrial features were filtered. Using Seurat (v5.3.0) and SCTransform, the data were normalized and transformed and the first 50 principal components were calculated and used for UMAP visualization. Cell clusters were identified based on a sweep of Louvain clustering resolutions and cluster marker genes were defined based on a Wilcoxon rank sum test as implemented in Seurat’s FindAllMarkers function. Clusters were annotated based on a combination of manual review and single-cell multiresolution marker-based annotation (scMRMA)^85^. After identifying immune cell subclusters, additional iterative rounds of normalization, dimensionality reduction, and cluster annotations was performed. For various groups of cells, we identified differentially expressed genes and conducted GSEA between peposertib and RT conditions as described above.

### Single-nucleus RNA sequencing of human glioblastoma samples

Nuclei were isolated from fresh frozen tumors a using a dounce tissue grinder in 2mL of EZ PREP lysis buffer (Sigma, #NUC-101). Nuclei suspensions were incubated on ice for 5 min, centrifuged at 500g for 5 min, and incubated and centrifuged a second time. Nuclei were washed once with nuclei suspension buffer (NSB) comprised of 1X PBS, 0.01% BSA, and 0.01% RNAse inhibitor (Clontech Cat#2313A), centrifuged at 500g for 5 min, resuspended in NSB, filtered through a 35μm cell strainer, and counted using nuclei staining with Hoechst 33342. Nuclei suspended in NSB were then processed according to the 10X Chromium Next GEM Single Cell 3’ GEM, Library & Gel Bead Kit v3.1 and sequenced on a NovaSeq X (Illumina). Library demultiplexing, alignment to the human genome GRCh38 reference, and UMI quantification was performed in CellRanger (10x Genomics). Published single nuclei data from glioblastoma before and after standard-of-care treatment was obtained from GEO accession GSE174554.

Data were processed using the Seurat R package (v5.3.0) and nuclei with greater than 200 genes were retained for downstream analysis. Data were transformed using SCTransform, UMAP was performed on the first 30 principal components, Louvain clustering was performed with resolution parameter of 0.4, and cluster identities were determined through (1) Enrichr analysis of the top 25 cluster marker genes as obtained using the FindAllMarkers function in Seurat, (2) single-cell multiresolution marker-based annotation (scMRMA)^85^, and (3) cross referencing to existing glioblastoma cell marker genes^6^. Tumor cells clusters were validated using InferCNV (v1.24) using the following parameters: cutoff = 0.1, max_cells_per_group = 100. Immune, glial, and neuronal cell clusters that were defined based on cluster markers were used as references for InferCNV. Differential gene set enrichment analysis was performed by first calculating differential gene expression for each individual gene using the DElegate (v1.1.0)^32,82^ implementation of DESeq2 (v2.1) against single transcriptomes pseudobulked into triplicates according to newly diagnosed or recurrent sample type. Gene set enrichment analysis was then performed on ranked lists derived from DESeq2 using fgsea (v1.27.1) with the following parameters: maxSize = 500, nPermSimple = 10000, separately for enriched and suppressed genes. A combined catalog of 2687 gene sets was aggregated from Reactome, MSigDB, and published glioblastoma marker genes^6,86^ and used as query genesets for fgsea.

### Spatial transcriptomics of human glioblastoma samples

Spatial transcriptomic profiling was performed on FFPE slides using the 10x Genomics Visium Spatial assay (10x Genomics, #1000336). Tissue was transferred to the capture areas on Visium glass slides using the 10x Genomics CytAssist, deparaffinized, and stained with hematoxylin and eosin (H&E). Libraries were prepared according to manufacturer instructions at the UCSF Genomics CoLab and sequenced on a Novaseq X (Illumina). Sequencing was performed with the recommended protocol (read 1: 28 cycles, i7 index read: 10 cycles, i5 index read: 10 cycles, read 2: 91 cycles). FASTQ sequencing files and histology images were processed using the 10x SpaceRanger pipeline and the Visium Human Transcriptome Probe Set v1.0 GRCh38–2020-A. Data were processed using the Seurat R package (v5.3.0), transformed using SCTransform, and UMAP was performed on the first 30 principal components. Louvain clustering was performed with resolution parameter of 0.3. Cluster identities were determined through Enrichr analysis of top 25 cluster markers obtained by the FindAllMarkers function in Seurat and cross referenced to existing glioblastoma marker genes^6^. Gene expression module analysis was performed using the MSigDB Hallmark genesets. Expression profiles along spatial trajectories were generated using SPATA^87^ (v2.3) in samples showing measurable areas of perivascular stroma (i.e. 218144, 218149). Euclidean distances were calculated for all spatial spots and the nearest spot corresponding to perivascular stroma. Expression scores were normalized to the maximum value for each gene module within each sample. Normalized expression scores were then aggregated for all samples and plotted as the mean ± standard error of the mean as a function of the distance to the perivascular border.

### Immunofluorescence imaging of cell cultures

Cells were seeded in Nunc Lab-Tek Cc2 8-well Chamber Slides (Thermo Fisher) or 15mm cover slips at a density of 50,000–75,000 cells per 200μL. Two hours after seeding, cells were treated with either 400nM peposertib or DMSO (vehicle control). One hour after treatment, cells were treated to 0, 2, 4, or 8Gy of ionizing radiation using the X-RAD 320 kV cabinet irradiator (Precision X-Ray). After a 24h incubation, cells were fixed with 4% PFA for 10 minutes, permeabilized in 0.1% Triton X-100 and TBS for 15 minutes, and blocked in 10% donkey serum and TBS for 2 hours. Cells were incubated in a primary antibody solution of 1:100 γH2AX Ser139 (Cell Signaling Technologies, #9718), 1:100 dsDNA antibody 35I9 DNA (Abcam, #ab27156), overnight at 4°C. Cells were washed in PBST and incubated in a secondary antibody solution of 1:1000 anti-rabbit IgG Alexa Fluor 568 (Thermo Fisher, #A10042) or 1:500 anti-mouse IgG Alexa Fluor 568 (Thermo Fisher, #A10037), and 1:1000 Hoechst 33342. Phalloidin was detected using conjugated 1:800 Alexa Fluor 488 Phalloidin (Cell Signaling, #8878S). Slides were incubated for 2 hours at room temperature in a dark, humidified chamber, then washed twice with PBST, and once with PBS. Coverslips were then mounted using VECTASHIELD Antifade Mounting Medium (Vector Laboratories) and sealed with nail polish. Slides were imaged on a Zeiss LSM800 confocal laser scanning microscope with Airyscan. Tiled images were taken at 40X using Immersol 518F, and processed using FIJI/ImageJ. Micronuclei and nuclei were quantified using the Stardist extension on Qupath. γH2AX foci were quantified using the built-in cell detection and subcellular detection features in Qupath. Cytoplasmic dsDNA was quantified using CellProfiler. Individual cytoplasmic areas were segmented based on phalloidin staining, with subtraction of nuclei. The area of dsDNA within those bounds was then quantified in microns squared.

### Multiplexed sequential immunofluorescence

Multiplexed sequential immunofluorescence was performed on the COMET platform (Lunaphore). FFPE tissue sections at 4μm thickness were mounted on charged Super Frost Plus microscope slides (ThermoFisher) prior to staining. An adjacent H&E tissue section was used to confirm tumor presence and location prior to staining. Unstained FFPE slides were processed for antigen retrieval using the PT Module (Epredia) with Dewax and HIER Buffer H (Lunaphore, #AR02) for 90 minutes at 102°C. Thereafter, slides were submerged in Multistaining Buffer (Lunaphore, #BU06). All primary antibodies were paired with a corresponding secondary antibody based on species: Alexa Fluor 555 (ThermoFisher, #A32727, dilution 1/200) and Alexa Fluor 647 (ThermoFisher, #A32733, dilution 1/400). The following primary antibodies were used for human specimens: GFAP (Sigma, #MAB360, dilution 1/3000), CD31 (Abcam, #Ab225883, dilution 1/1500), Ki67 (Agilent, #GA62661–2, dilution 1/1), CD206 (Abcam, #Ab64693, dilution 1/1000), STING (Cell Signaling, #13647S, dilution 1/100), and pIRF3 (Cell Signaling, #4947S, dilution 1/1500). The following primary antibodies were used for murine specimens: GFAP (Sigma, #MAB360, dilution 1/3000), CD31 (Abcam, #Ab225883, dilution 1/1500), Ki67 (Agilent, #GA62661–2, dilution 1/1), γH2AX (Abcam, #Ab26350, dilution 1/4000), STING (Cell Signaling, #13647S, dilution 1/100), and pIRF3 (Cell Signaling, #4947S, dilution 1/1500). All antibodies were diluted in Multistaining Buffer. Lastly, a DAPI counterstain was used for nuclear staining (ThermoFisher, #62248, dilution 1/1500). Each staining cycle consisted of a 4 minute period for primary antibodies and a 2 minute period for secondary antibodies. After each staining cycle, imaging was performed at 20x magnification using Imaging Buffer (Lunaphore, #BU09) to capture the TRITC and Cy5 dye signal. A subsequent 2 minute elution step was performed with Elution Buffer (Lunaphore, #BU07-L) with an additional 30 second quenching step utilizing Quenching Buffer (Lunaphore, #BU08-L). Slide imaging produced a multi-stack OME-TIFF file where the imaging outputs from each cycle were stitched and aligned. The OME-TIFF files contain a DAPI image autofluorescence signal via TRITC and Cy5 channels, and a single fluorescent layer per marker. Subsequent analysis was performed using the HORIZON Viewer software (Lunaphore). Prior to image analysis, background subtraction accounting for autofluorescence signal was performed and the threshold was set manually for each marker for snapshot generation. Quantification of positive cells for each marker was completed manually based on tumor segmentation.

### Cytoplasmic cellular fractionation

1×10^6^ cells were harvested in triplicate cultures 24 hours after treatment with peposertib and RT. Cells were fractionated using NE-PER Nuclear and Cytoplasmic Extraction Reagent (Thermo Fisher, #78833), and dsDNA within the cytoplasmic fractions were quantified using the SpectraMax Quant AccuClear Nano dsDNA Assay Kit (Molecular Devices, #R8357).

### Statistics

All experiments were performed with independent biological replicates and repeated as indicated. Statistics were derived from independent biological replicates, which are indicated in each figure panel or figure legend. Investigators were blinded to conditions during bioinformatic and functional analyses. Cell cultures and mice harboring PDXs were randomized to experimental conditions. No clinical, molecular, or cellular data points were excluded from the analyses. Data distributions were analyzed for normality prior to statistical testing. Results were compared using Student’s t-test, Mann-Whitney test, and other statistical approaches as indicated in the figure legends. In general, statistical significance is shown by asterisks and defined in the figure legends.

## Supplementary Material

Supplementary Files

This is a list of supplementary files associated with this preprint. Click to download.

- LiuEDFigv7.docx
- LiuNatCancerSupplementaryTablesv7.xlsx

## Data Availability

All computational code is deposited at https://github.com/liuj-lab/human_gbm_dnapk_public.

## Data availability

High throughput sequencing data have been deposited in SRA accession PRJNA1332306.
